# Triethanolamine-activated imine-linked covalent organic frameworks for highly efficient NADH generation

**DOI:** 10.1039/d6sc01309k

**Published:** 2026-03-16

**Authors:** Xiaoyu Li, Rui Liu, Han Cao, Chuanyin Tang, Guancheng Hua, Yingxu Hu, Xiangjiang Fan, Yongqing Xia, Shengjie Wang

**Affiliations:** a College of Chemistry and Chemical Engineering, China University of Petroleum Qingdao 266580 P. R. China sjwang@upc.edu.cn

## Abstract

Conjugated covalent organic frameworks (COFs) integrating different aromatic building units into extended π-conjugated backbones through imine linkages exhibit great potential in photocatalysis but suffer from lower efficiency of intramolecular electron transfer. Herein, we present a new strategy for the reversible activation of imine-linked COFs by triethanolamine (TEOA) under light irradiation. The activated COFs exhibit expanded solar light absorption, elevated reduction potential, decreased chemical impedance, suppressed recombination of charges, and significantly enhanced photocatalytic performance in the generation of nicotinamide adenine dinucleotide (NADH) without any metal cocatalysts. Experimental and theoretical results indicate that the fascinating photocatalytic performance originates from the protonation of imine bonds in the COFs, in which TEOA provides protons in addition to electrons, while light irradiation provides the driving force to overcome the energy barriers. This breaks through the traditional views that imine bonds can only be protonated under acidic conditions and provides new perspectives for the design of metal-free photocatalysts for highly efficient energy conversion.

## Abstract 2

We present a reversible activation mechanism of imine-linked COFs under non-acidic conditions with triethanolamine under light irradiation, in which TEOA functions as both an electron and proton donor.

## Graphical abstract

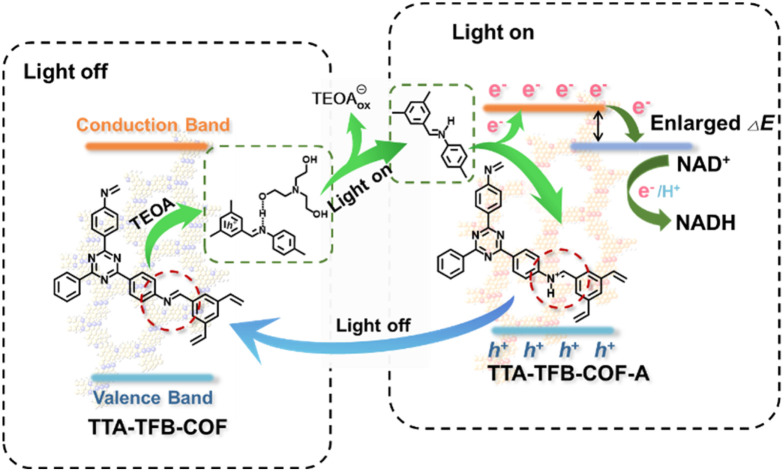

## Introduction

Photocatalysis utilizing solar light irradiation to generate electric/chemical energy or synthesize value-added compounds from harmful pollutants provides a viable strategy for mitigating the escalating energy and environmental problems. Organic, inorganic and metal materials have been used to catalyze such conversions.^1–6^ By contrast, covalent organic frameworks (COFs) composed of photosensitive ligands show great advantages in photocatalysis due to their structural regularity and stability, large surface area, tunable electronic structure, and tailorable functionality.^7,8^ In addition to light responsive organic ligands such as porphyrin,^9^ phthalocyanin,^10^ trazine,^11^ and other polycyclic compounds^12,13^ being used to absorb light and generate charges, the introduction of borate, triazine or imine linkages in such photosensitive COFs extends π-conjugations and allows charge transfer between the linked building units.^9,14^ Notably, imine linkages outperform alternative dynamic bonds in photocatalysis by functioning as sp^2^-hybridized building blocks that seamlessly integrate into π-extended frameworks, thereby interlinking conjugation pathways.^15^ However, highly polarized imine bonds are less favourable for charge transfer across them and limit their photocatalytic performance.^16,17^

Many efforts have been devoted to tackling this challenge.^18^ One effective method is used to improve the efficiency of charge separation by introducing electron donor–acceptor (D–A) pairs and an internal electric field into photosensitive COFs.^9,19–21^ Another method is used to optimize the charge transfer pathway by shortening the charge transfer distance or constructing individual transfer channels for electrons and holes within π-stacked columnar arrays.^7,22,23^ Additionally, post-synthesis modification of imine linkages provides a flexible strategy to address the poor efficiency of charge transfer and integrates certain novel functionalities into imine-linked COFs.^15,24–26^ Particularly, protonation of imine bonds demonstrates significant advantages in expanding light absorption, improving charge transfer efficiency, enhancing photocatalytic activity, and altering reaction pathways.^12,21,26–29^ However, such protonation only occurred under acidic conditions restricted to the p*K*_a_ values of imine bonds (pH 5–7).^26,29^ How to activate the imine-linked COFs in non-acidic environments emerges as a scientifically imperative yet challenging task.

**Scheme 1:**
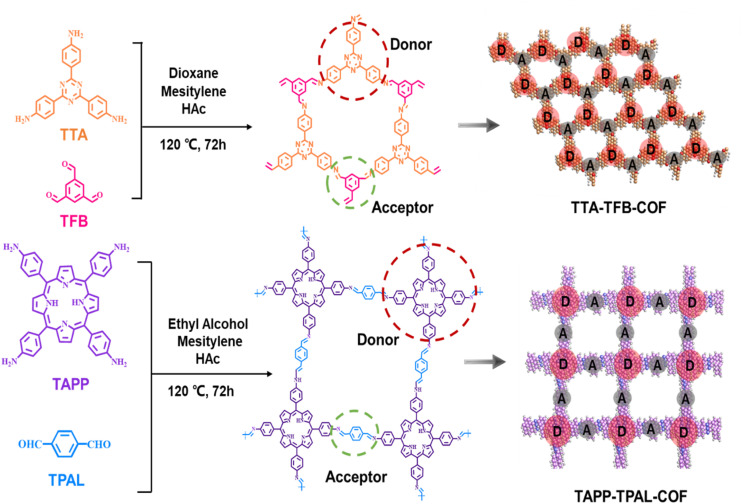
Schematic illustration of the building units and synthetic process of TTA–TFB-COF and TAPP–TPAL-COF.

Porphyrin- and triazine-based COFs with D–A structures have been reported, and their conjugated structures and wide application in photocatalysis attract great attention.^21,26,30^ Herein, we present a novel approach to obtain high-performance photocatalysts by activating imine-linked COFs under non-acidic conditions. Porphyrin- (TAPP–TPAL-COF) and triazine-based (TTA–TFB-COF) COFs with electron donor–acceptor structures were synthesized by a Schiff base reaction (Scheme 1). As a photocatalyst, TTA–TFB-COF was activated and exhibited expanded light absorption, elevated reduction potential, improved efficiency in charge transfer and superior photocatalytic capability in NADH generation, although TAPP–TPAL-COF showed higher light absorption capacity. Experimental and theoretical evidence demonstrated the activation mechanism. The imine bonds in TTA–TFB-COF are protonated by TEOA under light irradiation. TEOA not only functions as a sacrificial electron donor but also as a proton donor, while light irradiation provides the driving force to overcome the energy barriers. This work presents a novel method to activate imine-linked COFs under non-acidic conditions, and demonstrates the protonation mechanism of imine bonds by the cooperation of TEOA and light irradiation.

## Results and discussion

### Synthesis and characterization of D–A COFs

**Fig. 1:**
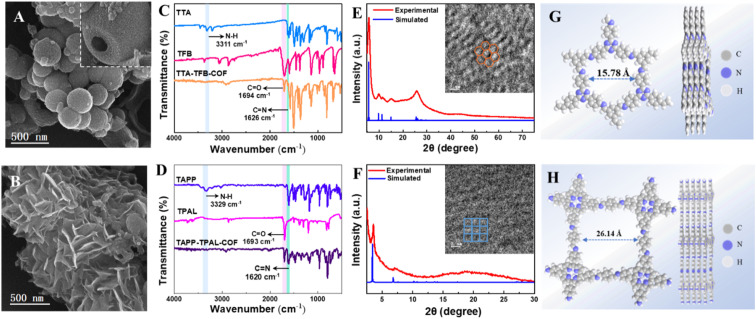
SEM images (A and B), FT-IR spectra (C and D), and PXRD patterns (E and F) of TTA–TFB-COF (A, C, and E) and TAPP–TPAL-COF (B, D and F). The inset in image (A) shows hollow spheres for TTA–TFB-COF. The insets in images (E) and (F) show the HRTEM images of TTA–TFB-COF and TAPP–TPAL-COF. Top and side views of the simulated packing structure of TTA–TFB-COF (G) and TAPP–TPAL-COF (H).

Triazine- (TTA–TFB-COF) and porphyrin-based (TAPP–TPAL-COF) COFs were fabricated using Schiff base reactions (Scheme 1). Molecular orbital positions in imine-linked COFs were determined using density functional theory (Fig. S1). The highest occupied molecular orbitals (HOMO) and the lowest unoccupied molecular orbitals (LUMO) were occupied by TTA and TFB in TTA–TFB-COF, respectively. As for TAPP–TPAL-COF, the HOMO and LUMO were occupied by TAPP and TPAL, respectively. This suggests that both triazine- and porphyrin-based COFs are typical D–A systems, in which TTA or TAPP function as electron donors, while TFB or TPAL act as electron acceptors, respectively.^31^ Spatial isolation between the HOMO and LUMO would facilitate charge separation and transfer in D–A COFs. SEM images (Fig. 1A and B) show hollow spheres with diameters of ∼300 nm for TTA–TFB-COF, while stacked flakes with a thickness of ∼5 nm for TAPP–TPAL-COF. As shown in the FTIR spectra of the synthesized materials (Fig. 1C and D), the disappearance of peaks at 3300–3400 cm^−1^ (amino groups), remarkable decrease in peak intensity at 1690–1695 cm^−1^ (aldehyde groups), and appearance of new peaks at 1620–1630 cm^−1^ (imine linkages) demonstrated the formation of imine-linked COFs by the condensation of amino and aldehyde groups.^26,29^ Moreover, the appearance of the peak at 399.2 eV in the N 1s and 286.1 eV in the C 1s X-ray photoelectric spectra (XPS) of TTA–TFB-COF, and 399.0 eV in the N 1s spectrum and 285.7 eV in the C 1s spectrum of TAPP–TPAL-COF further confirmed the formation of imine bonds (Fig. S2 and S3).

Powder X-ray diffraction (PXRD) measurements confirmed the excellent crystallinity of the synthesized COFs (Fig. 1E and F). The PXRD data for TTA–TFB-COF showed sharp diffraction peaks at 5.6°, 9.8°, 11.4°, 14.9° and 25.7°, which originated from the diffraction of (100), (210), (230), (340) and (001) planes, respectively, consistent with the simulated result.^29^ The density-functional tight-binding (DFTB) method, including Lenard-Jones (LJ) dispersion, was used to optimize the sheet conformation stacking structure.^32^ A hexagonal structure with an AA stacking model was proposed for TTA–TFB-COF (Fig. 1G). In comparison, the PXRD spectrum of TAPP–TPAL-COF also shows a sharp peak in the low-angle region (3.2° (100)), consistent with the simulated data, but fitting a square lattice model (Fig. 1H). HR-TEM images (the inset images in Fig. 1E, F and S4) further verified the hexagonal structure for TTA–TFB-COF and square morphology for TAPP–TPAL-COF, respectively. The variations in topological structure may affect the charge transfer and photocatalytic performance of the COFs.

### Band structure of D–A COFs

TAPP–TPAL-COF displayed strong absorption across 200–800 nm in UV-DRS spectra, whereas TTA–TFB-COF mainly exhibited light responsiveness in the ultraviolet region (Fig. S5A). Accordingly, their Tauc plots indicated that TAPP–TPAL-COF possessed a narrower band gap (Fig. S5B and C). Their flat band potentials (*E*_fb_) were obtained from the intercepts of the Mott–Schottky plots (Fig. S5D and E). For n-type semiconductors, their conduction band edge (*E*_CB_) is generally close to the *E*_fb_.^33,34^ Therefore, the conduction band potential (*E*_CB_) could be replaced by the *E*_fb,_ and the band structures of the COFs were obtained according to *E*_g_ = *E*_CB_ + *E*_VB_ (Fig. S5F). The results indicated that the two D–A COFs had similar reduction potentials, but TAPP–TPAL-COF showed a narrower band gap.

### Photocatalytic production of NADH

**Fig. 2:**
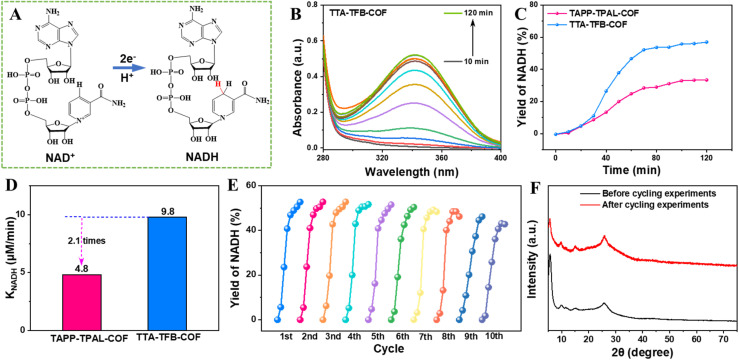
Schematic illustrating the production of NADH from NAD^+^ by capturing one proton and two electrons (A). UV-vis spectra of the reaction system for NADH production under light irradiation using TTA–TFB-COF as the photocatalyst and TEOA as the sacrificial electron donor (B). Plots showing increases in the yield of NADH with time over TTA–TFB-COF and TAPP–TPAL-COF photocatalysts (C). Photocatalytic kinetic constants for NADH production over different photocatalysts (D). Cycling experiments for photocatalytic generation of NADH over TTA–TFB-COF (E). PXRD patterns over the TTA–TFB-COF photocatalyst before and after five-cycle photocatalytic experiments (F).

The photocatalytic ability of the prepared D–A COFs was evaluated by the generation of NADH. As a coenzyme, NADH is critical for numerous biochemical reactions and artificial photosynthesis. However, its high cost and photocatalytic generation relying heavily on noble-metal co-catalysts (*e.g.*, Pt nanoclusters or Rh complexes) elevate regeneration expenses and complicate subsequent purification.^35–38^ Consequently, noble-metal-free photocatalysts for NADH production are critically needed. The fabricated COFs drive the reduction of NAD^+^ to NADH through capturing one proton and two electrons from TEOA in the absence of metal co-catalysts (Fig. 2A). The production of NADH was tracked *via* the absorbance at 340 nm in the UV-vis spectra (Fig. 2B, S6 and S7). The products were further confirmed by high-performance liquid chromatography (HPLC) with the detection wavelength at 340 nm (Fig. S8). The peak at 0.88 min belonging to the NADH increased with the reaction time, in line with the results shown in the UV-vis spectra.

The TTA–TFB-COF exhibited high efficiency with a NADH yield of 56.9% (Fig. 2C), higher than that of TAPP–TPAL-COF (33.4%) and most of the reported metal-free photocatalysts (Table S1). In addition, the photocatalytic turnover frequency (TOF) of TTA–TFB-COF reached 2.85 mmol g^−1^ h^−1^, which is also greater than that of TAPP–TPAL-COF (1.67 mmol g^−1^ h^−1^). The production rate of NADH over the TTA–TFB-COF photocatalyst was 9.8 µM min^−1^, 2.1 times that over TAPP–TPAL-COF (Fig. 2D). Cycling experiments show that the yield of NADH has a slight decrease after ten cycles (Fig. 2E and S9A), indicating that the D–A COFs have excellent cycle stability and reusability. Additionally, unchanged crystal structures of the recycled photocatalysts (Fig. 2F and S9B) further demonstrated their excellent stability. The results indicate promising scalability of the method, such as high stability and reproducibility in short-term tests, which are encouraging. As for industrial applications, COF materials still face certain challenges in controlling the crystallinity and complex synthesis processes, which currently hinder their large-scale production and practical implementation.

Significantly, TTA–TFB-COF exhibited astonishing photocatalytic performance, although it possessed a narrower light absorption spectrum and relatively larger size compared to TAPP–TAPL-COF. The intrinsic mechanism for the remarkable photocatalytic activity of TTA–TFB-COF deserves intensive study.

### Mechanism analysis

**Fig. 3:**
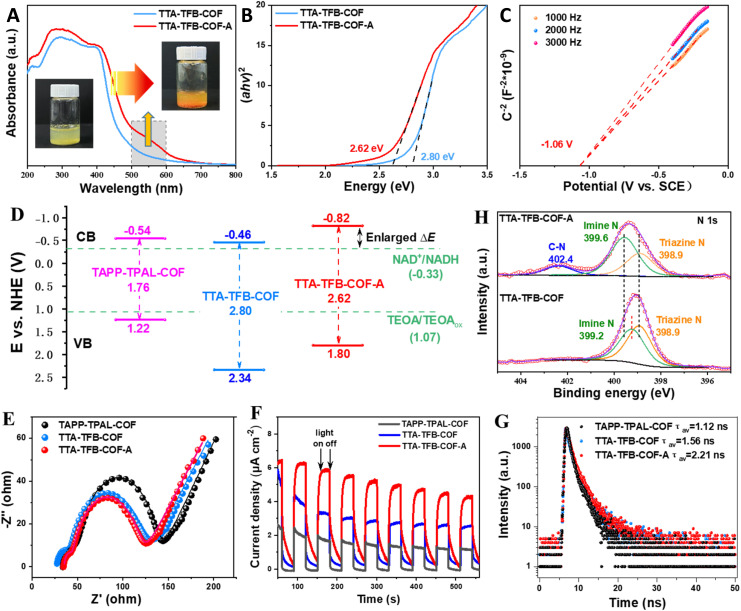
UV-DRS spectra ((A), insets: optical photographs), Tauc plots (B), Mott–Schottky plots (C), energy band structures (D), electrochemical impedance patterns (E), photocurrent curves (F), transient photoluminescence spectra (G), and high-resolution N 1s XPS spectra (H) of TTA–TFB-COF solution before and after light irradiation (labeled as TTA–TFB-COF-A). For comparison, the energy band structure, electrochemical impedance, photocurrent, and transient photoluminescence spectrum of TAPP–TPAL-COF were also involved in panels (D)–(G).

An interesting phenomenon was observed during the photocatalytic generation of NADH, in which the color of the TTA–TFB-COF photocatalyst changed from yellow to orange (Fig. 3A and S10) under light irradiation (it reverted to the original color when the photocatalyst was kept in the dark for ∼2 hours), while no detectable color change was found for the TAPP–TPAL-COF system. Accompanied by the color change of TTA–TFB-COF, its absorbance spectrum extended to the long wavelength direction (Fig. 3A). The Tauc plots (Fig. 3B) and Mott–Schottky patterns (Fig. 3C) confirmed the change in the energy band structure of TTA–TFB-COF during the reaction process. As shown in Fig. 3D, the band gap of the photocatalyst decreased, but the conduction band position shifted upward, confirming its improved solar light utilization efficiency and enhanced reduction potential compared to the pristine TTA–TFB-COF. This indicated that the TTA–TFB-COF was activated during the reaction process. For convenience of expression, the activated photocatalyst was denoted as TTA–TFB-COF-A in the following section.

To unveil the intrinsic mechanism for the remarkably improved photocatalytic performance of TTA–TFB-COF-A, the photoelectric properties of the synthesized COFs were systematically investigated. The electrochemical impedance spectroscopy results revealed the smallest radius of TTA–TFB-COF-A, indicating that charge transfer within the electrode and electrolyte was accelerated (Fig. 3E).^39^ The increased photocurrent density for TTA–TFB-COF-A further demonstrated its superior charge separation performance (Fig. 3F).^5,40^ Additionally, time-resolved photoluminescence spectroscopy probed the charge carrier dynamics (Fig. 3G). The average lifetimes of TAPP–TPAL-COF, TTA–TFB-COF, and TTA–TFB-COF-A were 1.12 ns, 1.56 ns, and 2.21 ns, respectively. A longer lifetime for an active species means a slower recombination rate and a higher probability to participate in the photocatalytic reaction.^41,42^ This may be another important factor to guarantee the fascinating photocatalytic properties of TTA–TFB-COF-A.

The changes in color, photoelectric properties, and photocatalytic performance of TTA–TFB-COF-A conform to protonated imine-linked COFs, which are usually generated under acidic conditions.^21,26,29,43^ However, in the present work, the activation occurred at pH ∼10.0, greater than the p*K*_a_ of imine bonds (pH 5–7)^12^ and depended on TEOA and light irradiation, suggesting that a novel activation mechanism should be involved. The color of the TTA–TFB-COF photocatalyst became black (Fig. S11) when ascorbic acid (CA) was used as the electron donor, similar to previous reports,^44^ but no NADH generation occurred. Neither color change nor NADH generation could be observed when TEOA was replaced by triethylamine (TEA), diethanolamine (DEOA), and ethanolamine (EOA) (Fig. S10), suggesting the specific function of the terminal hydroxyl groups and tertiary structure of TEOA in the activation process.

**Fig. 4:**
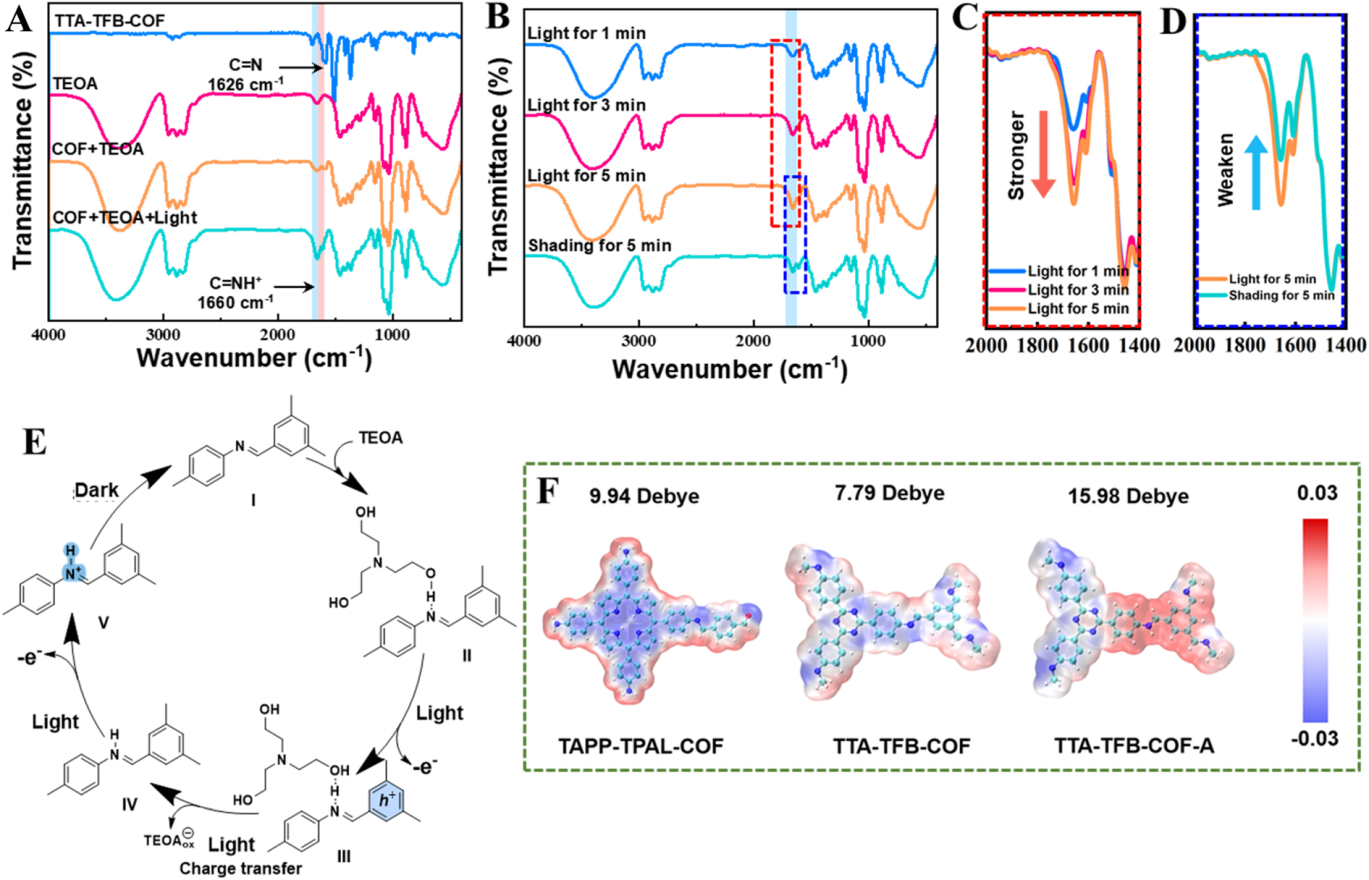
FTIR spectra of TTA–TFB-COF, TEOA, TTA–TFB-COF/TEOA, and TTA–TFB-COF/TEOA under light irradiation (A). FTIR spectra of the reaction system with different irradiation times and then returning the mixture to dark conditions (B). Magnified views of the key spectral regions from panel (C) and (D). Schematic showing the proposed activation of TTA–TFB-COF by TEOA under light irradiation (E). The molecular dipolar and electron distribution of TAPP–TPAL-COF, TTA–TFB-COF, and TTA–TFB-COF-A (F).

The activation process was monitored by the XPS spectra (Fig. 3H). Compared to the N 1s XPS spectra of the pristine TTA–TFB-COF, the binding energy of the imine N atoms of TTA–TFB-COF-A showed a redshift of ∼0.4 eV, while the triazine N atoms showed no change. This indicated that the electron density around the imine N atoms decreased during the activation process, consistent with the protonation of imine bonds where the electrons of imine N atoms migrated to the electron-deficient protons.^26–28^ The protons for the protonation of imine bonds may come from the sacrificial electron donor (TEOA) since the conversion occurred under alkaline conditions, and the protonation is driven by light irradiation to overcome the dynamics barrier. Fourier transform infrared (FT-IR) spectroscopy provided further confirmation of imine bond protonation. A comparison of FT-IR spectra before and after light irradiation revealed the emergence of a new peak at 1660 cm^−1^ (Fig. 4A), which is attributed to the stretching vibration of protonated imine bonds (C=NH^+^).^26,44^ It was found that the intensity of the peak at 1660 cm^−1^ gradually increased with prolonged irradiation time (Fig. 4B and C) by monitoring the spectral changes over different light irradiation times, indicating that light irradiation promotes the protonation of imine bonds. The peak intensity at 1660 cm^−1^ decreased significantly when the sample was subsequently treated under dark conditions (Fig. 4B and D), suggesting deprotonation of the protonated imine bonds. These results demonstrate that the protonation process is driven by light irradiation and is reversibly suppressed under dark conditions, exhibiting clear photo-responsive reversible characteristics. Undoubtedly, this would shorten the charge transfer distance from the sacrificial electron donor to the photocatalyst and contribute to the photocatalytic efficiency.

Considering the above evidence, the TEOA-activated process of TTA–TFB-COF could be summarized in Fig. 4E. (I) TEOA molecules adsorb on the surface of the TTA–TFB-COF photocatalyst *via* hydrogen bonding when they are mixed. (II) Electrons transfer from TTA to TFB under light irradiation, and positive holes are left in TTA. (III) The positive holes are neutralized by the photoinduced electrons from TEOA, and at the same time, TEOA is oxidized. (IV) Imine bonds are protonated by capturing the hydrogen of hydroxyl groups in TEOA under light irradiation. (V) The protonated imine bonds are thermodynamically unstable and slowly neutralized by the hydroxyl ions in solution once the photocatalysts are isolated from light irradiation.

Moreover, D–A configurations in the fabricated COFs generate internal electric fields, which promote charge separation *via* propelling photoinduced electrons and holes toward opposite directions.^45,46^ A series of density functional theory (DFT) computations probed its influence on charge transfer kinetics, as the internal electric field correlates with surface charge density and dipole moments. Given the internal electric field's linkage to surface charge density and molecular dipole,^47,48^ TAPP–TPAL-COF, TTA–TFB-COF, and TTA–TFB-COF-A underwent DFT simulation of electron distribution along with molecular dipole moments. Fig. 4F reveals that positive charges were localized at the protonated imine linkages, as determined from the surface electrostatic potential calculated using the monomeric model. Based on rigorous analysis, we also calculated the surface electrostatic potential using a periodic structure model (Fig. S12). The results obtained from the periodic structure and those from the monomeric model are nearly identical and largely consistent. The mutual validation of the two approaches confirms that the derived surface electrostatic potential is reliable. TTA–TFB-COF-A showed the largest molecular dipole moment, indicating that its protonation promotes the special separation of charges and facilitates the establishment of a larger internal electric field, consistent with the experimental results.

**Fig. 5:**
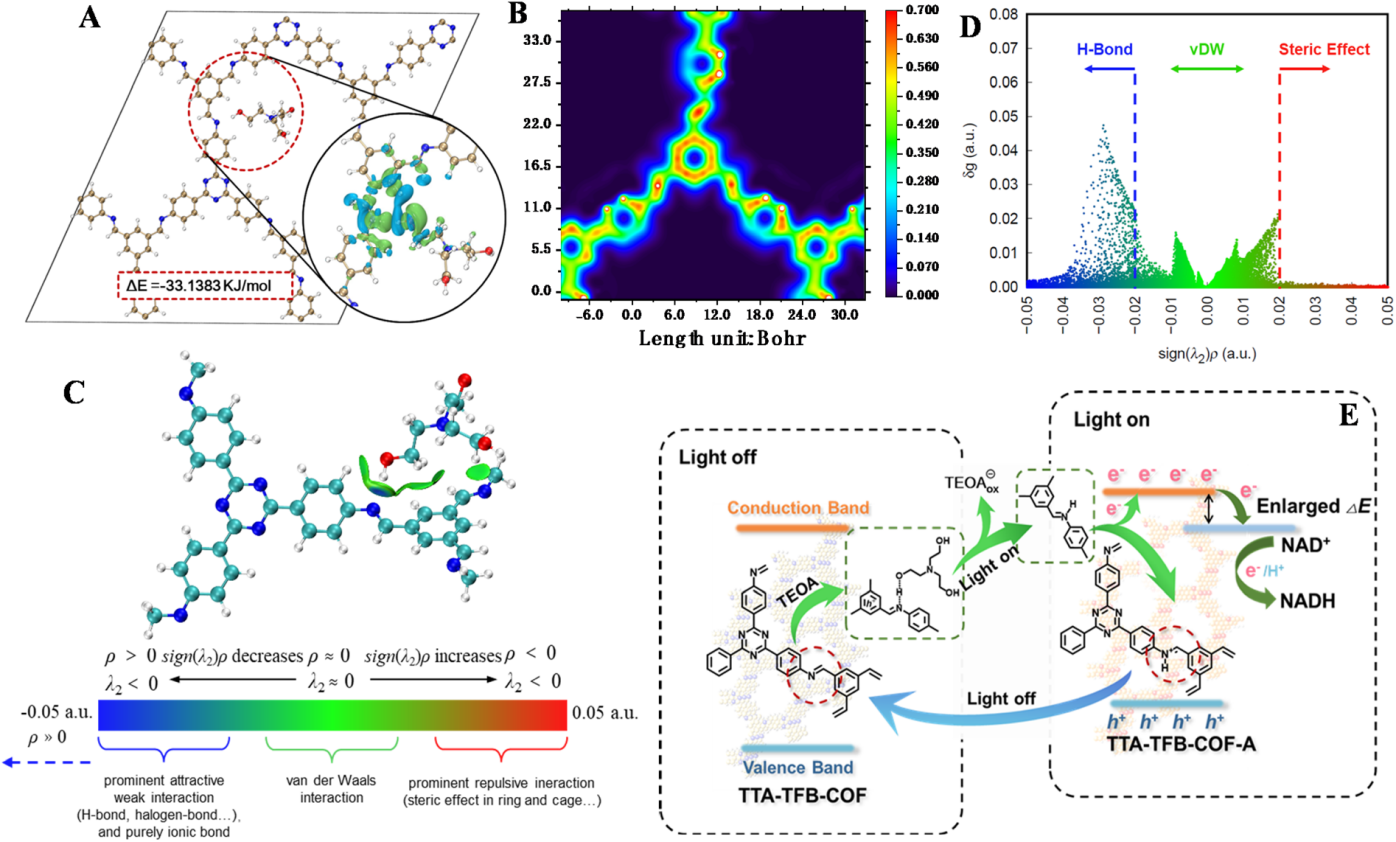
DFT calculations. Density difference charge map of triethanolamine combined with TTA–TFB-COF (A). Visualization of TTA–TFB-COF π conjugation intensity (B). The IGMH analysis of TTA–TFB-COF and TEOA (C) and the IGMH scatter plot of TTA–TFB-COF and TEOA (D). The activation mechanism of the imine-linked COF for NADH generation (E).

To simulate the interaction of the COF with TEOA, a periodic structural model containing TEOA was constructed. Differential charge density analysis (Fig. 5A) reveals an increase in electron density in the protonated imine bond regions of the COF, accompanied by a decrease around the hydroxyl hydrogen atoms of TEOA, confirming interfacial charge transfer. To quantify the adsorption strength, the adsorption energy (Δ*E*) was subsequently evaluated using the TZV2P-MOLOPT-PBE-GTH basis set and GTH pseudopotentials. The adsorption energy is defined as Δ*E* = *E*_tot_ − *E*_COF_ − *E*_mol_, where *E*_tot_ is the total energy of the adsorbate–substrate complex, and *E*_COF_ and *E*_mol_ are the energies of the isolated substrate and isolated adsorbate, respectively. The calculated Δ*E* values are −33.1383 kJ mol^−1^ for COF-TEOA and −30.5182 kJ mol^−1^ for COF–H_2_O (Fig. 5A and S13). More negative Δ*E* values correspond to greater adsorption stability, confirming that TEOA forms a more stable complex with the COF than water does. Furthermore, π–π interactions were examined using the Localized Orbital Locator (LOL-π) method (Fig. S14), and the π-electron conjugation strength was visualized (Fig. 5B). Independent gradient model based on Hirshfeld partition (IGMH) analysis further identifies hydrogen bonding as the predominant interaction between TEOA and the COF, which is characterized by a distinct peak in the region corresponding to sign(*λ*_2_)*ρ* < 0 in the two-dimensional scatter plot (Fig. 5C and D).^49^ Based on the above computational results, it can be concluded that TEOA binds to the imine linkage site of TTA–TFB-COF *via* hydrogen bonding, accompanied by notable charge transfer. The ability of TEOA to form a more stable complex with the COF suggests that the hydrogen used in the protonation of the imine bond is likely provided by TEOA.

The marked difference demonstrates the capability of TTA–TFB-COF-A in enhancing electron transfer and charge separation, thereby accounting for the high photocatalytic efficiency of TTA–TFB-COF in NADH production (Fig. 5E). (1) The protonation of imine-linked COF occurs in the presence of TEOA under light irradiation, which reduces the polarity of the linkage and endows the COFs with a narrowed band gap, elevated conduction band, and enlarged internal electric field; (2) TEOA functions not only as a sacrificial electron donator, but also as a proton donor; (3) the protonation is reversible, depending on light irradiation or not.

## Conclusions

In summary, we synthesized triazine- and porphyrin-based COFs with D–A structures to tackle the challenge of imine-linked COFs in charge transfer. The triazine-based COFs can be activated by TEOA under light irradiation and show an extraordinary photocatalytic activity and cycle stability. A yield of 56.9% for NADH generation was obtained with a reaction rate of 9.8 µmol min^−1^ in the absence of any noble metals, greater than that of most metal-free photocatalysts. The activation mechanism of the imine-linked COFs was disclosed from experimental and theoretical evidence. Imine bonds in TTA–TFB-COF accept protons from TEOA in addition to electrons under light irradiation and result in its protonation, which brings a wide light absorption range, high reduction potential, longer lifetimes of active species, enhanced charge transfer efficiency, and fascinating photocatalytic performance. This work elucidates the activation mechanism of imine-linked COFs under non-acidic environments, offering novel perspectives for their photocatalytic design and applications.

## Author contributions

Xiaoyu Li: data curation; investigation; writing – original draft, Rui Liu: investigation; visualization; writing – original draft, Han Cao: investigation – simulation, Chuanyin Tang: investigation – supporting, Guancheng Hua: investigation – supporting, Yingxu Hu: investigation – supporting, Xiangjiang Fan: investigation – supporting, Yongqing Xia: investigation – supporting, Shengjie Wang: conceptualization; data curation; formal analysis; funding acquisition; methodology; project administration; resources; supervision; writing – review & editing.

## Conflicts of interest

There are no conflicts to declare.

## Supplementary Material

SC-017-D6SC01309K-s001

## Data Availability

The data supporting this article have been included as part of the supplementary information (SI). Additional data are available from the corresponding author on reasonable request. Supplementary information: experimental methods (showing the synthesis of imine-linked COFs, photocatalytic generation of NADH, characterization methods); Table S1 (comparison of photocatalytic ability in NADH generation); Fig. S1–S14 (showing DFT simulated molecular orbital, XPS spectra, TEM and HRTEM images, UV-DRS and Mott–Schottky plots, UV-vis spectra, HPLC spectra, cycle experiments, XRD and FTIR spectra) and additional references. See DOI: https://doi.org/10.1039/d6sc01309k.

## References

[cit1] Ham R., Nielsen C. J., Pullen S., Reek J. N. H. (2023). Chem. Rev..

[cit2] Xiu L., Zhang L., Du X., Cao H., Wang D., Hu Y., Qiao Y., Ma Y., Xia Y., Wang S. (2024). ACS Sustainable Chem. Eng..

[cit3] Wi D. H., Yang H., Kim Y., Ahn H., Hong J. W., Han S. W. (2023). J. Mater. Chem. A.

[cit4] Zhang Y., Tian J., Shaikh H., MacKenzie H. K., He Y., Zhao C., Lei S., Ren Y., Manners I. (2023). J. Am. Chem. Soc..

[cit5] Liu F., Cao H., Xu L., Fu H., Sun S., Xiao Z., Sun C., Long X., Xia Y., Wang S. (2023). J. Colloid Interface Sci..

[cit6] Tang C., Li X., Hu Y., Du X., Wang S., Chen B., Wang S. (2024). Molecules.

[cit7] Zhao S.-S., Liang J., Si D.-H., Mao M.-J., Huang Y.-B., Cao R. (2023). Appl. Catal., B.

[cit8] Li Z., Dong Z., Zhang Z., Wei B., Meng C., Zhai W., Wang Y., Cao X., Han B., Liu Y. (2025). Angew. Chem., Int. Ed..

[cit9] Li X., Tang C., Zhang L., Song M., Zhang Y., Wang S. (2023). Biomimetics.

[cit10] Matheu R., Gutierrez-Puebla E., Monge M. A., Diercks C. S., Kang J., Prévot M. S., Pei X. K., Hanikel N., Zhang B., Yang P. D., Yaghi O. M. (2019). J. Am. Chem. Soc..

[cit11] Xu L. Q., Ding S. Y., Liu J. M., Sun J. L., Wang W., Zheng Q. Y. (2016). Chem. Commun..

[cit12] Gu Y.-H., Xu X., Yuan S. (2025). Chem.–Eur. J..

[cit13] Liu W., Zhang Y. N., Wang J., Shang X. B., Zhang C. X., Wang Q. L. (2024). Coord. Chem. Rev..

[cit14] Yang X., Xu Q., Wei W., Zeng G. (2025). Angew. Chem., Int. Ed..

[cit15] Li X. L., Zhang C. L., Cai S. L., Lei X. H., Altoe V., Hong F., Urban J. J., Ciston J., Chan E. M., Liu Y. (2018). Nat. Commun..

[cit16] Rao M. R., Fang Y., De Feyter S., Perepichka D. F. (2017). J. Am. Chem. Soc..

[cit17] Guan X., Qian Y., Zhang X., Jiang H.-L. (2023). Angew. Chem., Int. Ed..

[cit18] Qian C., Feng L. L., Teo W. L., Liu J. W., Zhou W., Wang D. D., Zhao Y. L. (2022). Nat. Rev. Chem..

[cit19] Shan H. C., Cai D., Zhang X. X., Zhu Q., Qin P. Y., Baeyens J. (2022). Chem. Eng. J..

[cit20] Kim Y. H., Jeon J. P., Kim Y., Noh H. J., Seo J. M., Kim J., Lee G., Baek J. B. (2023). Angew. Chem., Int. Ed..

[cit21] Yang J., Acharjya A., Ye M. Y., Rabeah J., Li S., Kochovski Z., Youk S., Roeser J., Grüneberg J., Penschke C., Schwarze M., Wang T. Y., Lu Y., van de Krol R., Oschatz M., Schomäcker R., Saalfrank P., Thomas A. (2021). Angew. Chem., Int. Ed..

[cit22] Zhu Y., Shao P., Hu L., Sun C., Li J., Feng X., Wang B. (2021). J. Am. Chem. Soc..

[cit23] Joshi T., Chen C., Li H. F., Diercks C. S., Wang G. Q., Waller P. J., Li H., Bredas J. L., Yaghi O. M., Crommie M. F. (2019). Adv. Mater..

[cit24] Zou L., Sa R. J., Zhong H., Lv H. W., Wang X. C., Wang R. H. (2022). ACS Catal..

[cit25] Lyu H., Li H., Hanikel N., Wang K., Yaghi O. M. (2022). J. Am. Chem. Soc..

[cit26] Dong P. F., Xu X. Y., Wu T. K., Luo R. G., Kong W. S., Xu Z. Y., Yuan S., Zhou J., Lei J. P. (2024). Angew. Chem., Int. Ed..

[cit27] Che Q., Li C. Y., Chen Z. H., Yang S., Zhang W. F., Yu G. (2024). Angew. Chem., Int. Ed..

[cit28] Tan H. Q., Kang Z. H., Li Y. G., Qiu T. Y., Zhao Y. N., Tang W. S., Sun H. Y., Zhao X. (2022). ACS Catal..

[cit29] Liu X. L., Yang X. Y., Ding X., Wang H. L., Cao W., Jin Y. C., Yu B. Q., Jiang J. Z. (2023). Chin. Chem. Lett..

[cit30] Zhao F., Xu F., García H., Yu J. (2025). J. Colloid Interface Sci..

[cit31] Zhi Y. F., Ma S., Xia H., Zhang Y. M., Shi Z., Mu Y., Liu X. M. (2019). Appl. Catal., B.

[cit32] Chen X., Addicoat M., Jin E. Q., Xu H., Hayashi T., Xu F., Huang N., Irle S., Jiang D. L. (2015). Sci. Rep..

[cit33] Tong H., He R., Chen G., Tong Z., Dang M., Li J., Wu D., Qian D. (2024). J. Colloid Interface Sci..

[cit34] Tang C., Cao H., Gao J., Wang S., Liu R., Chen B., Si Q., Xia Y., Wang S. (2025). J. Colloid Interface Sci..

[cit35] Hu Y. X., Peng J. F., Liu R., Gao J., Hua G. C., Fan X. J., Wang S. J. (2024). Molecules.

[cit36] Wang S., Li M., Patil A. J., Sun S., Tian L., Zhang D., Cao M., Mann S. (2017). J. Mater. Chem. A.

[cit37] Xiu Y., Xu L., Zhang X., Wang X., Liu F., Xia Y., Cao M., Wang S. (2020). ACS Sustainable Chem. Eng..

[cit38] Kim J. H., Lee M., Lee J. S., Park C. B. (2012). Angew. Chem., Int. Ed..

[cit39] Cao H., Liu F., Tai Y., Wang W., Li X., Li P., Zhao H., Xia Y., Wang S. (2023). Chem. Eng. J..

[cit40] Xiu Y., Zhang X., Feng Y., Wei R., Wang S., Xia Y., Cao M., Wang S. (2020). Nanoscale.

[cit41] Ma J. N., Miao T. J., Tang J. W. (2022). Chem. Soc. Rev..

[cit42] Guo L., Niu Y., Razzaque S., Tan B., Jin S. (2019). ACS Catal..

[cit43] Yang J., Acharjya A., Ye M.-Y., Rabeah J., Li S., Kochovski Z., Youk S., Roeser J., Grüneberg J., Penschke C., Schwarze M., Wang T., Lu Y., van de Krol R., Oschatz M., Schomäcker R., Saalfrank P., Thomas A. (2021). Angew. Chem., Int. Ed..

[cit44] Ascherl L., Evans E. W., Gorman J., Orsborne S., Bessinger D., Bein T., Friend R. H., Auras F. (2019). J. Am. Chem. Soc..

[cit45] Zhao X., Shang S., Liu H., Peng C., Hu J. (2024). Chemosphere.

[cit46] Yue X., Fan J., Xiang Q. (2022). Adv. Funct. Mater..

[cit47] Yang H., Zhao R., Wang J., Yin X., Lu Z., Hou L. (2023). Acs Mater. Let..

[cit48] Li J., Cai L., Shang J., Yu Y., Zhang L. (2016). Adv. Mater..

[cit49] Lu T., Chen Q. (2022). J. Comput. Chem..

