# Supplementary material for: Triethanolamine-activated imine-linked covalent organic frameworks for highly efficient NADH generation
Source: Chem Sci. 2026 Mar 16;17(17):8787–94. doi: 10.1039/d6sc01309k (PMC12989824; doi:10.1039/d6sc01309k)
Supplement: SC-017-D6SC01309K-s001 [file SC-017-D6SC01309K-s001.pdf]

Supplementary Information (SI) for Chemical Science.
This journal is © The Royal Society of Chemistry 2026

Supporting Information

Triethanolamine-activated imine-linked covalent organic frameworks for highly efficient NADH generation

Xiaoyu Li†, Rui Liu†, Han Cao, Chuanyin Tang, Guancheng Hua, Yingxu Hu, Xiangjiang Fan, Yongqing Xia, Shengjie Wang*

College of Chemistry and Chemical Engineeriny, China University of Petroleum (East China), Qingdao, 266580 People's Republic of China.

† Xiaoyu Li and Rui Liu contributed equally.
** Please contact Dr. Shengjie Wang: sjwang@upc.edu.cn*

Abstract: Conjugated covalent organic frameworks (COFs) integrating different aromatic building units into extended π-conjugated backbones by imine linkages exhibit great potential in photocatalysis but suffer from lower efficiency of intramolecular electron transfer. Herein, we present a new strategy for the reversible activation of imine-linked COFs by triethanolamine (TEOA) under light irradiation. The activated COFs exhibit expanded solar light absorption, elevated reduction potential, decreased chemical impedance, suppressed recombination of charges, and significantly enhanced photocatalytic performance in the generation of nicotinamide adenine dinucleotide (NADH) without any metal cocatalysts. Experimental and theoretical evidence indicate that the fascinating photocatalytic performance originates from the protonation of imine bonds in the COFs, in which TEOA provides protons in addition to electrons, while light irradiation provides the driving force to overcome the energy barriers. This breaks through the traditional views that imine bonds can only be protonated at acidic conditions and provides new perspectives for the design of metal-free photocatalysts for highly efficient energy conversion.

1. Chemicals

2,4,6-Tris(4-aminophenyl)-1,3,5-triazine (TTA, > 98% in purity), 1,3,5-triformylbenzene (TFB, > 99% in purity), 5,10,15,20-Tetrakis(4-aminophenyl)porphyrin (TAPP, > 97% in purity), and terephthalaldehyde (TPAL, > 98% in purity) were provided by Jilin Yanshen Technology Co., Ltd. Nicotinamide adenine dinucleotide (NAD^+, > 96.5% in purity) and reduced nicotinamide adenine dinucleotide (NADH, > 97% in purity) were purchased from Sigma-Aldrich. Triethanolamine (TEOA, analytical grade), mesitylene (analytical grade), 1,4-dioxane (analytical grade), absolute alcohol, anhydrous tetrahydrofuran (THF), and glacial acetic acid were supported by Sinopharm Chemical Reagent Co., Ltd. All the chemicals were used as received. All water used was from a Millipore water purification system with a minimum resistivity of 18.2 MΩ.

2. Experimental methods

2.1 Synthesis of imine-linked COFs

Synthesis of TTA-TFB-COF: TTA (70 mg, 0.2 mmol) and TFB (48 mg, 0.2 mmol) were dissolved in a mixed solvent (10 mL, 1,4-dioxane (v): mesitylene (v) = 10:1) and sonicated for 15 min. Aqueous HOAc (0.75 mL, 6 M) was added to the mixture, transferred to a 25 mL Schlenk storage tube, and degassed by three freeze-pump-thaw cycles. The tube was sealed under vacuum and heated at 120 °C for 5 days to complete the reaction. Then the reaction system was cooled to room temperature, and yellow precipitates were collected through centrifugation, washing with anhydrous THF 3 times, and drying under vacuum at 60 °C for 12 h. The precipitates were labeled as TTA-TFB-COF.

Synthesis of TAPP-TPAL-COF: TAPP (13.70 mg，0.02 mmol) and TPAL (5.36 mg，0.04 mmol) were dissolved in a mixed solvent (2 mL, ethanol (v): mesitylene (v) = 1:1) and transferred to a 10 mL Schlenk storage tube, and sonicated for 15 min. Aqueous HOAc (0.2 mL, 6M) was added to the mixture and degassed by three freeze-pump-thaw cycles. The tube was sealed under vacuum and heated at 120 °C for 5 days to obtain a precipitate. Black purple precipitates were collected through centrifugation, washing with anhydrous THF 3 times, and drying under vacuum at 60 °C for 12 h. The precipitates were labeled as TAPP-TPAL-COF.

2.2 Photocatalytic generation of NADH

NADH was produced from its oxidation state (NAD^+) using TEOA as the sacrificial electron donor. In a typical reaction, 3 mg of photocatalyst, 300 μL of NAD^+ stock solution (10 mM), and 450 mg of TEOA were mixed in a transparent vial. A certain amount of ultrapure water was added to bring the volume to 3.0 mL. The mixture was then exposed to visible-light irradiation (a 300 W xenon lamp with a 400 nm cutoff filter, 85 mW cm^{-2}). Liquid samples were taken from the reaction mixture at certain intervals and centrifuged at 10,000 rpm for 5 min. The concentration of NADH in the supernatants was evaluated by monitoring the absorbance at 340 nm in the UV–vis spectra. The yield of NADH was calculated by Eq. 1.

$$Yield\ of\ NADH(\%) = \frac{C_{NADH}}{C_{NAD^+}} \times 100\% \qquad (1)$$

where C_{NADH} in the reaction system was calculated by the absorbance at 340 nm of the samples and the standard curve of the absorbance versus the concentration of NADH, where $C_{NAD}{}^+$ is equal to the initial concentration of NAD^+ (1 mM).

3. Characterization methods

Powder X-ray diffraction (XRD) was performed using an X Pert PRO MPD X-ray diffractometer. **Transmission electron microscope (TEM)** and **high-resolution TEM (HR-TEM)** images were collected using a JEOL 2100 UHR microscope and operated at 200 kV. **Scanning electron microscopy (SEM)** experiments were performed on a TESCAN MIRA LMS instrument with an accelerated voltage of 3 kV. **X-ray photoelectron spectra (XPS)** were measured on an ESCALAB 250 X-ray photoelectron spectrometer with a monochromatic X-ray source (Mg Kα, hν = 1253.6 eV). XPS measurements were also performed on a Thermo Scientific™ K-Alpha™+ spectrometer equipped with a monochromatic Helium Iα ultralight source (21.2 eV) to obtain the valence band maximum of the samples. **UV–vis absorption spectra** were recorded using a UV–vis spectrophotometer (UV2450, SHIMADZU). **The steady-state fluorescence spectra** of the solid samples were measured at 25 °C using a FluoroMax 4 (Horiba Jobin Yvon) spectrophotometer with an excitation wavelength of 320 nm. **The fluorescence lifetimes** of the samples were determined by a time-correlated single-photon counting technique (Edinburgh Instruments Spectrofluorometer FS5) equipped with a

405 nm diode laser. **UV-vis diffuse reflectance spectra (UV-DRS)** were obtained with a UV2450 spectrophotometer (SHIMADZU) equipped with an integrating sphere using $BaSO_4$ as the reference. **High-performance liquid chromatography (HPLC) analysis** was conducted on an EClassical 3100 series system from Elite Company. Separation was carried out on a SinoPak BEH T-C18 chromatographic column (5 μm) and operated at 30°C. The mobile phase consists of acetonitrile and water (25:75, v/v), and is conveyed at 1.0 mL min^{-1}. **Electrochemical measurements** of the samples were performed on an electrochemical workstation CHI 660E (Shanghai CH Instrument, China) at room temperature using a three-electrode system with an indium tin oxide (ITO) photoanode covered with samples as the working electrode, a platinum foil as the counter electrode, and a saturated calomel ($Hg/HgCl_2$) electrode as the reference electrode. A xenon lamp (300 W, CEL-HXUV300 with a 400 nm cut-off filter, 85 mW cm^{-2}) was used as the light source. **The transient photocurrent responses** and **Mott–Schottky plots** of the samples were obtained under visible light irradiation using Na_2SO_4 solution (0.5 mol L^{-1}) as the electrolyte. **The electrochemical impedance spectroscopy (EIS)** measurements were performed in the darkness by immersing the ITO glass electrode coated with samples into a phosphate buffer solution (pH 7, 100 mM) containing 5 mM of $K_3(CN)_6Fe$ and $K_4(CN)_6Fe$.

4. Calculation

Energy levels and frontier molecular orbital diagrams calculations: The optimized structures of TAPP-TPAL-COF, TTA-TFB-COF and TTA-TFB-COF-A have been performed on Gaussian 16 software. The B3LYP functional and def2SV basis sets were used for geometry optimization calculations. Wave function files were obtained from single-point calculations at B3LYP/631G (d, p) level to analyze the orbital composition and electrostatic potential by the Multiwfn program. The results were visualized with Visual Molecular Dynamics (VMD) 1.9.3 and VESTA.

First-principles density functional theory (DFT) calculations using the PBE functional were performed to investigate the adsorption characteristics of triethanolamine molecules on the TTA-TFB-COF surface. The minimum-energy structures considered in this work were first optimized with Grimme's D3 dispersion correction. The D3 method is a dispersion-correction

strategy proposed by Grimme and co-workers.[1, 2] It can be coupled with most DFT functionals and is applicable to all elements of the periodic table, spanning systems from molecules to solids. This approach improves the description of weak dispersion interactions with negligible additional computational cost, which is essential when noncovalent intermolecular interactions are expected to play a significant role. Structural optimizations and electronic-structure calculations were carried out using CP2K (version 2023.1) [3] with DZVP-MOLOPT-SR-GTH basis sets and Goedecker–Teter–Hutter (GTH) pseudopotentials. Subsequently, single-point energies (and adsorption energies) were evaluated using the TZV2P-MOLOPT-PBE-GTH basis set and GTH pseudopotentials. The adsorption energy (Ead), used to quantify adsorption strength, was defined as:

$\Delta E = E_{tot} - E_{COF} - E_{mol}$

where E_{tot} is the total energy of the adsorbate–substrate complex, and E_{COF} and E_{mol} are the energies of the isolated substrate and isolated adsorbate, respectively. More negative ΔE values indicate more stable adsorption configurations.

Independent Gradient Model based on Hirshfeld partition (IGMH): The Independent Gradient Model based on Hirshfeld partition (IGMH) [4] analysis was performed to investigate the non-covalent interactions within and between molecules. IGMH analysis was conducted using Multiwfn and visualized with VMD. (Colour mapping was applied based on sign $(\lambda_2)\rho$ to distinguish different types of interactions: Blue regions indicate strong attractive interactions (e.g., hydrogen bonding); Green regions represent weak van der Waals interactions; Red regions correspond to steric repulsion).Intramolecular electron delocalization, a pivotal theory for elucidating inter-interactions, describes the expansion and distribution of electron clouds throughout the entire molecular system. This delocalization reveals the strong electronic correlations established between different regions via delocalized electrons. LOL-π (localized orbital locator) can precisely probe into the intramolecular electron delocalization behaviors.

References

[1] Kocman, M.; Jurecka, P.; Dubecky, M.; Otyepka, M.; Cho, Y.; Kim, K. S. Phys. *Chem. Chem. Phys.*, 2015, **17**, 6423-6432.

[2] Grimme, S.; Antony, J.; Ehrlich, S.; Krieg, H. *J. Chem. Phys.* 2010, **132**, 154104.

[3] Kuehne, T. D.; Iannuzzi, M.; Del Ben, M.; Rybkin, V. V.; Seewald, P.; Stein, F.; Laino, T.; Khaliullin, R. Z.; Schutt, O.; Schiffmann, F.; et al. *J. Chem. Phys.,* 2020, **152**, 194103.

[4] T. Lu, Q. Chen, *J. Comput. Chem.* 2022, 43, 539-555.

Supporting Tables

Table S1. Comparison of the photocatalytic activity of various photocatalysts for NADH generation

Materials	Reduction	Spectral region	Electron mediator	Regeneration yield
T-COF-1[1]	NADH	300 W Xe Lamp (>420 nm)	$[Cp^*Rh(bpy)(H)]^+$	74%
TP-COF[2]	NADH	300 W Xe Lamp (>420 nm)	-	43.8%
nano-TP-TTA[3]	NADH	300 W Xe Lamp (>420 nm)	Cp*Rh complex	51.2%
NKCOF-113[4]	NADH	300 W Xe Lamp (>420 nm)	$[Cp^*Rh(bpy)(H)]^+$	58%
2D CTF[5]	NADH	300 W Xe Lamp (>420 nm)	Cp*Rh complex	75.88%
PTF-1h[6]	NADH	300 W Xe Lamp (>420 nm)	-	35.25%
DBBTS-CMP2	NADH	λ = 410 nm LED Lamp	$[CpRh(bpy)H_2O]^{2+}$	39% (in 45 min)
mpg-C_3N_4 [8]	NADH	λ = 420 nm LED Lamp	$[CpRh(bpy)H_2O]^{2+}$	10%
NU-1006[9]	NADH	White light irradiation	[CpRh(bpydc)Cl]	28%
TD+[Cp*Rh][10]	NADH	300 W Xe Lamp (>420 nm)	$[CpRh(bpy)H_2O]^{2+}$	38%
TTA-TFB-	NADH	300 W Xe Lamp (>400 nm)	-	56.9%

[1] Y. Wang, H. Liu, Q. Pan, C. Wu, W. Hao, J. Xu, R. Chen, J. Liu, Z. Li, Y. Zhao, *Journal of the American Chemical Society* **2020**, *142*, 5958-5963.
[2] Y. Zhao, H. Liu, C. Wu, Z. Zhang, Q. Pan, F. Hu, R. Wang, P. Li, X. Huang, Z. Li, *Angewandte Chemie International Edition* **2019**, *58*, 5376-5381.
[3] J. Liu, X. Ren, C. Li, M. Wang, H. Li, Q. Yang, *Applied Catalysis B: Environmental* **2022**, *310*, 121314.
[4] Z. Zhao, D. Zheng, M. Guo, J. Yu, S. Zhang, Z. Zhang, Y. Chen, *Angewandte Chemie International Edition* **2022**, *61*, e202200261.
[5] R. K. Yadav, A. Kumar, N.-J. Park, K.-J. Kong, J.-O. Baeg, *Journal of Materials Chemistry A* **2016**, *4*, 9413-9418.
[6] D. Yadav, A. Kumar, J. Y. Kim, N.-J. Park, J.-O. Baeg, *Journal of Materials Chemistry A* **2021**, *9*, 9573-9580.
[7] F. Lan, Q. Wang, H. Chen, Y. Chen, Y. Zhang, B. Huang, H. Liu, J. Liu, R. Li, *ACS Catalysis* **2020**, *10*, 12976-12986.
[8] J. Liu, M. Antonietti, *Energy & Environmental Science* **2013**, *6*, 1486-1493.
[9] Y. Chen, P. Li, J. Zhou, C. T. Buru, L. Đorđević, P. Li, X. Zhang, M. M. Cetin, J. F. Stoddart, S. I. Stupp, M. R. Wasielewski, O. K. Farha, *Journal of the American Chemical Society* **2020**, *142*, 1768-1773.
[10] H. Zheng, Z. Huang, P. Wei, Y. Lin, Y. Cao, X. Zhang, B. Zhou, C. Peng, *ACS Sustainable Chemistry & Engineering* **2025**, *13*, 4078-4092.

Supplementary Figures

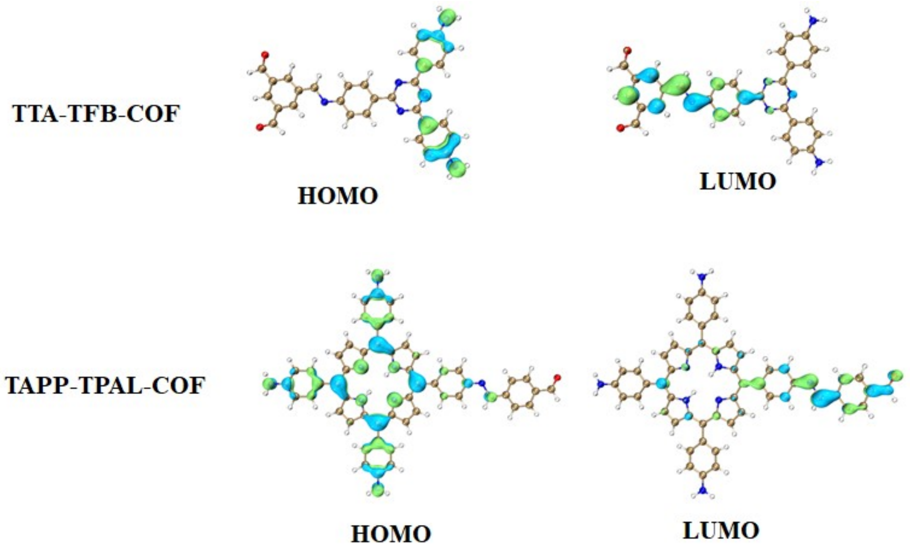

Figure S1. DFT simulated frontier molecular orbital of TTA-TFB-COF and TAPP-TPAL-COF. The highest occupied molecular orbitals (HOMO) were located in the TTA part and the lowest unoccupied molecular orbital (LUMO) in the TFB part for TTA-TFB-COF. As for TAPP-TPAL-COF, the HOMO was located in the TAPP while the LUMO in the TPAL part.

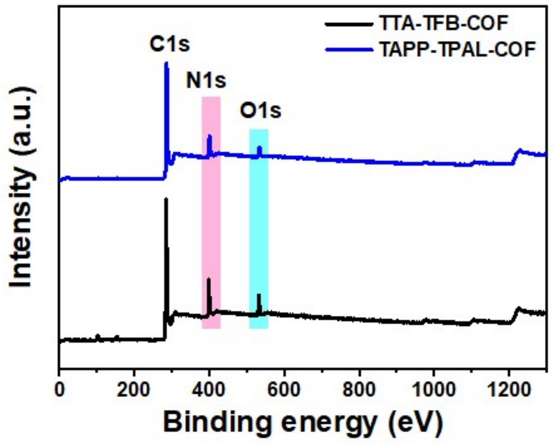

Figure S2. XPS survey spectra of TTA-TFB-COF and TAPP-TPAL-COF.

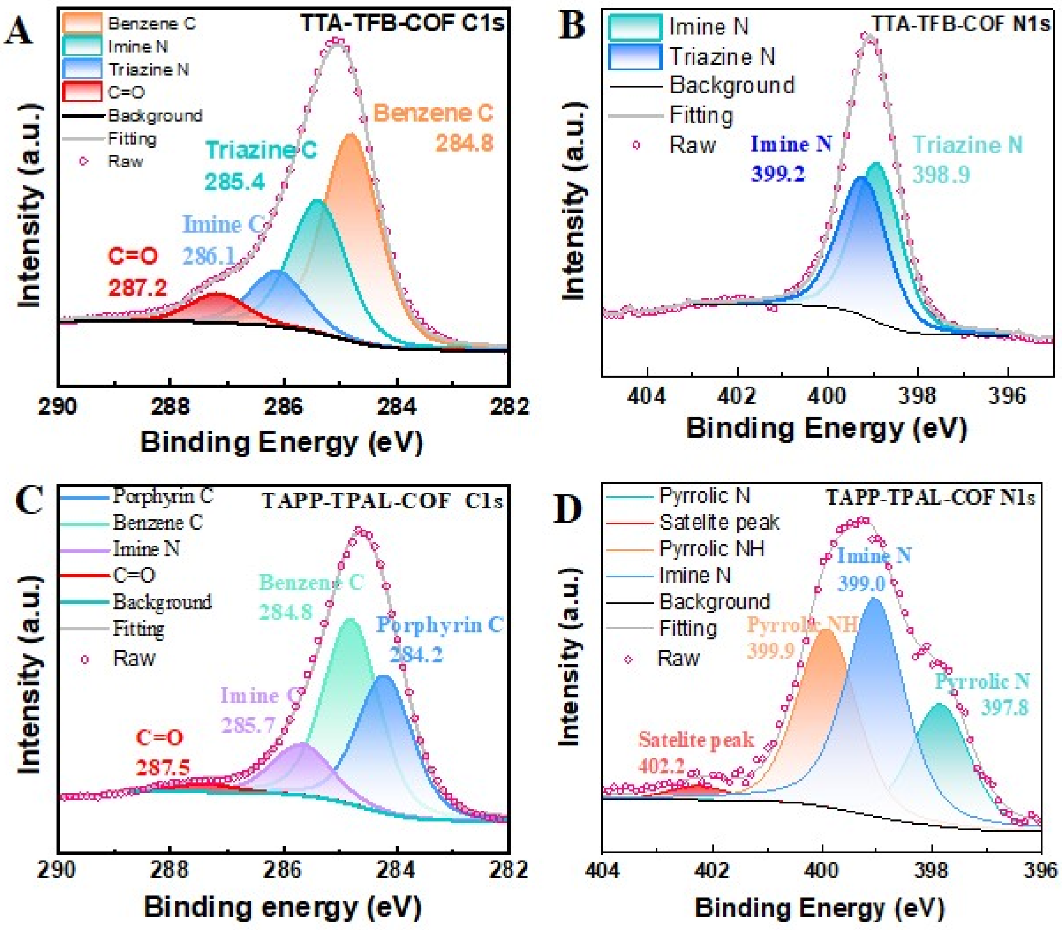

Figure S3. C 1s (A, C) and N 1s (B, D) high-resolution XPS spectra of TTA-TFB-COF (A, B) and TAPP-TPAL-COF (C, D).

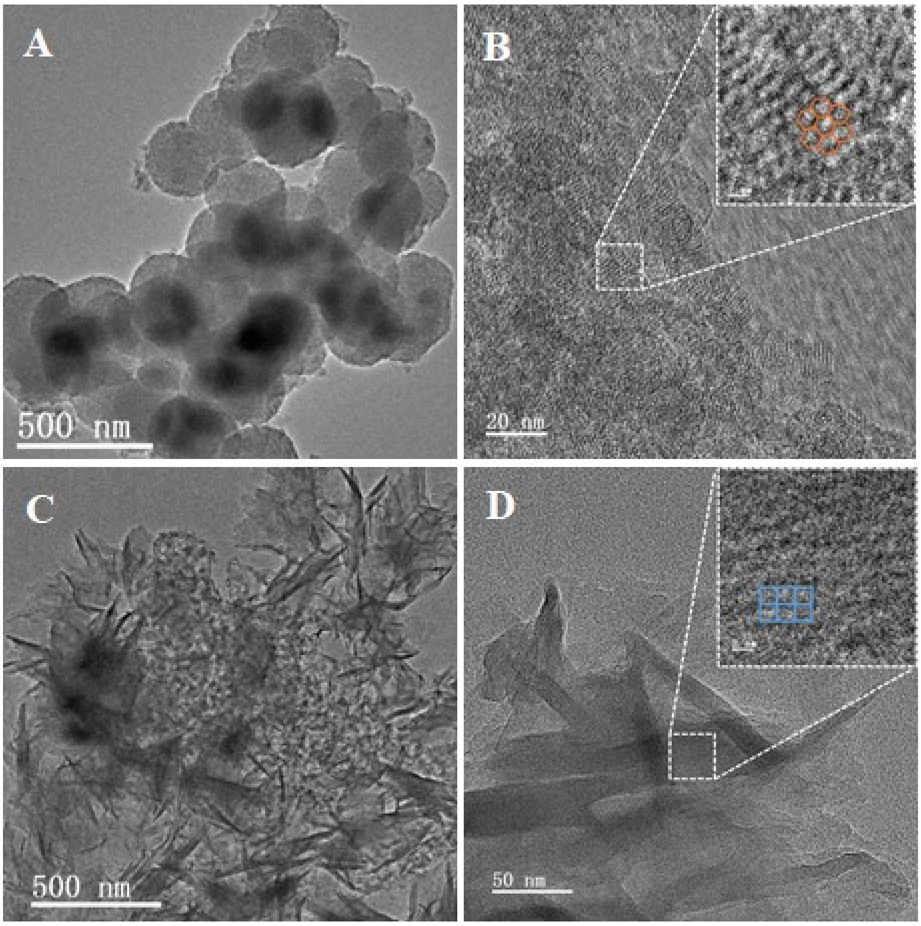

Figure S4. TEM and HR-TEM images of TTA-TFB-COF (A, B) and TAPP-TPAL-COF (C, D). The insets in image B and D were their corresponding lattice fringe images, which suggested that TTA-TFB-COF exhibited a hexagonal while TAPP-TPAL-COF showed a quadrilateral topological structure, respectively.

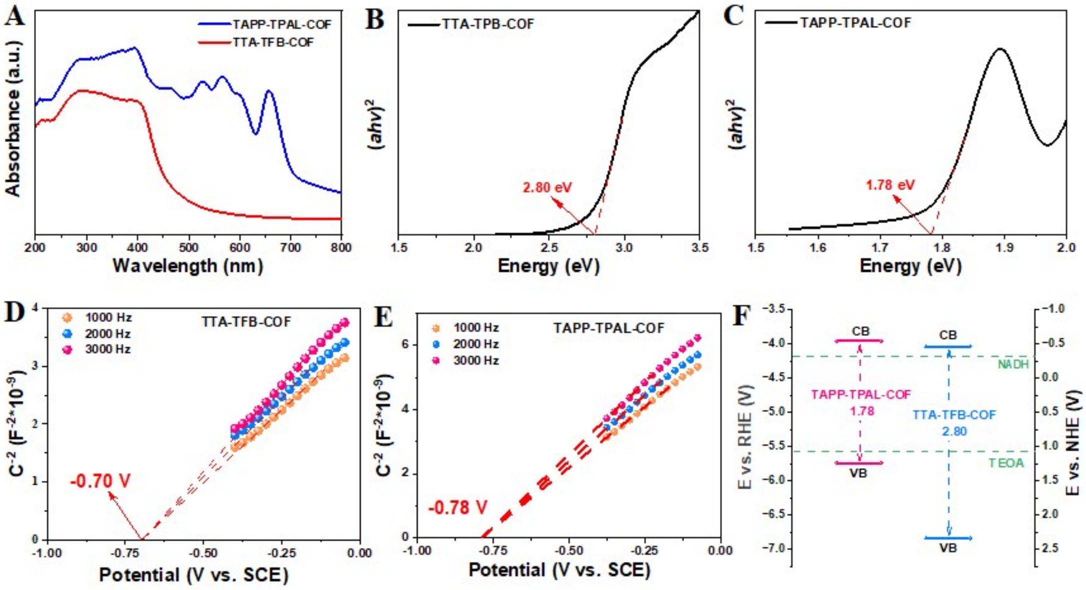

Figure S5. UV-vis diffuse reflectance spectra (A), Taut plots (B, C), Mott-Schottky plots (D, E) and band structure (F) of TTA-TFB-COF and TAPP-TPAL-COF.

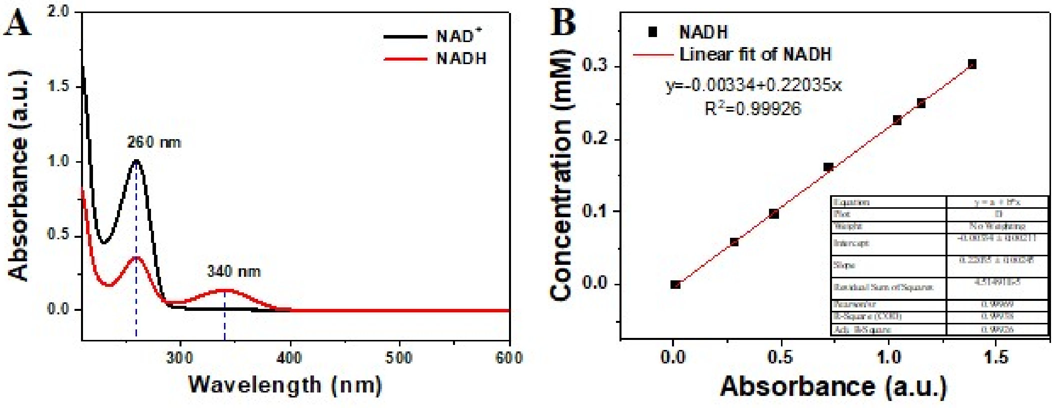

Figure S6. UV-Vis spectra of NAD^+ and NADH (A). Standard working curves of the concentration of NADH versus its absorbance at 340 nm in the UV-vis spectra (B).

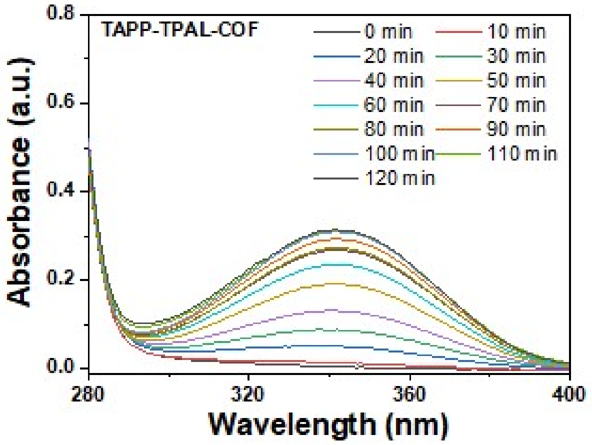

Figure S7. UV-vis spectra of the NADH generation system using TAPP-TPAL-COF as the photocatalyst and TEOA as the sacrificial electron donor exposed to light irradiation.

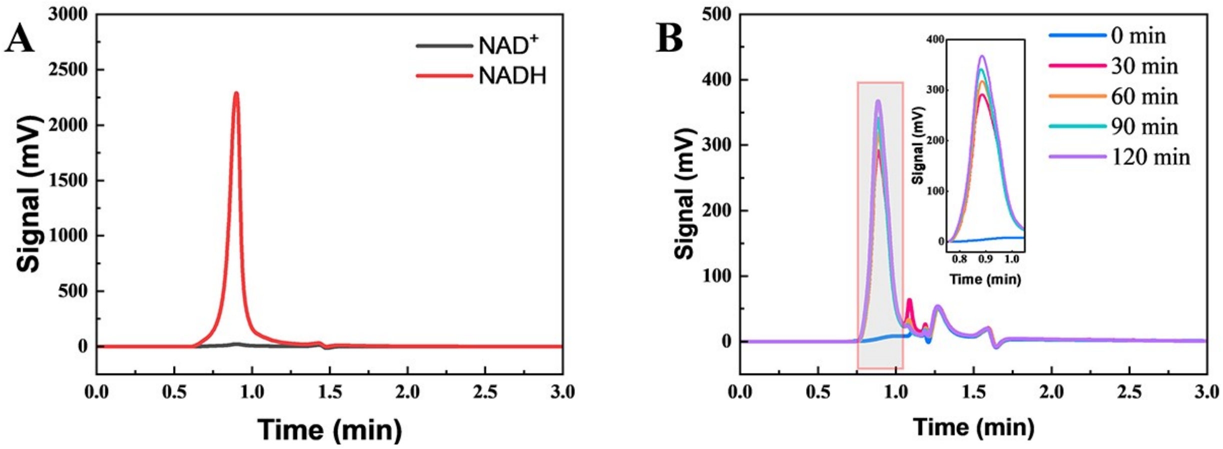

Figure S8. HPLC spectra of NAD^+ and NADH. Using an acetonitrile-to-water ratio of 1:1 (v/v) as the mobile phase and a detection wavelength of 340 nm, NADH eluted as a standard peak at 0.88 min, whereas NAD^+ did not show any detectable signal. (B) HPLC spectra of the reaction system with various reaction times.

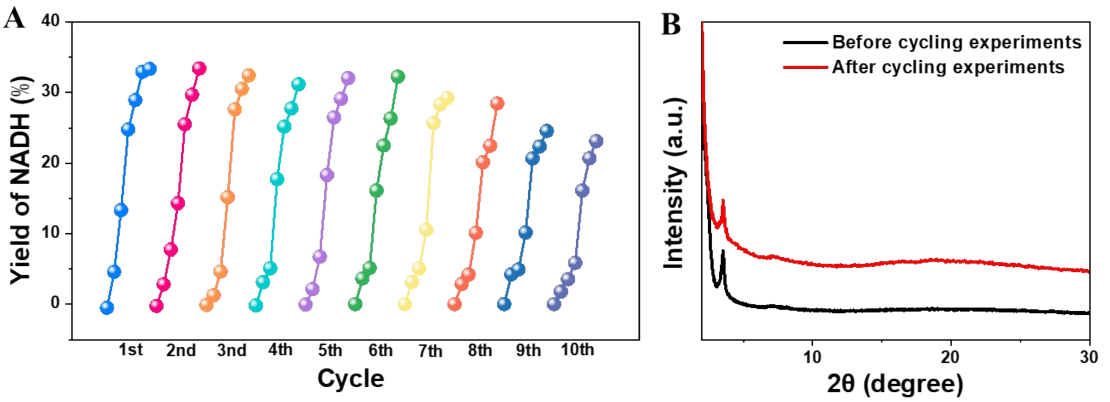

Figure S9. Yields of NADH with irradiation time in the presence of the recycled TAPP-TPAL-COF photocatalyst (A). XRD patterns of the TAPP-TPAL-COF photocatalyst before and after 5 cycling experiments (B).

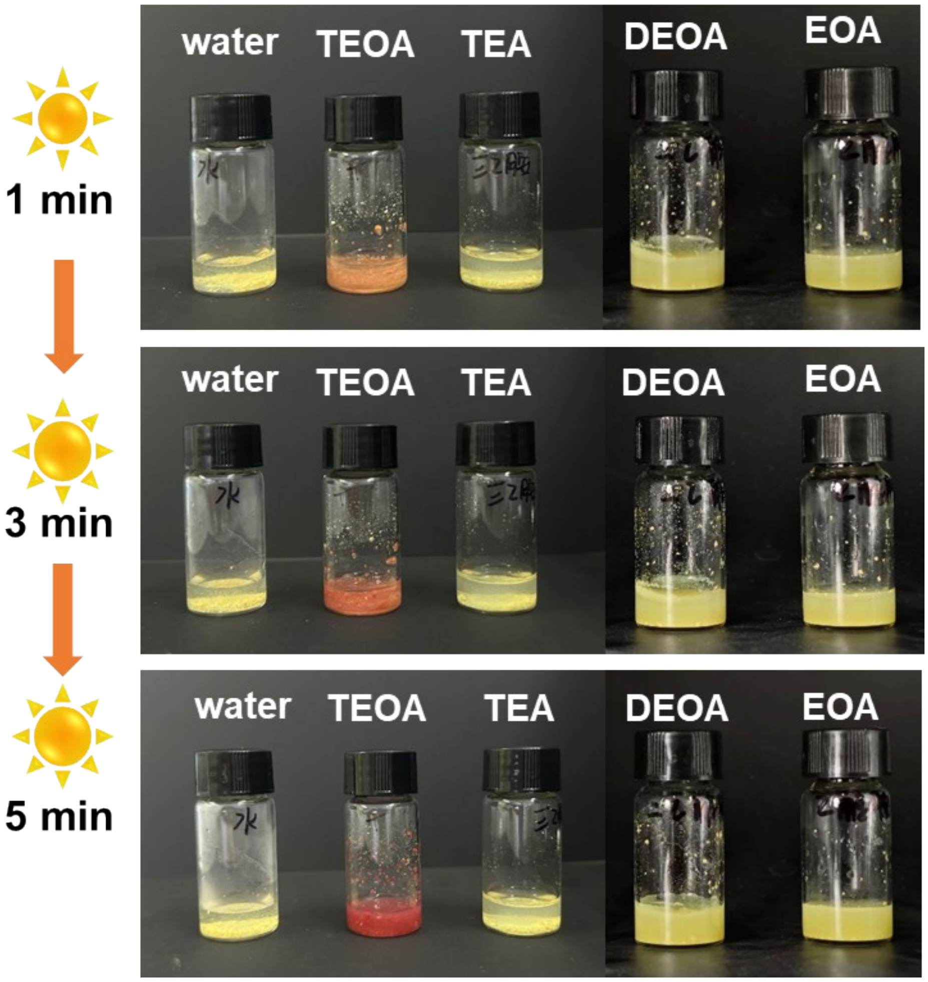

Figure S10. Photography images of the TTA-TFB-COF photocatalyst in water, triethanolamine (TEOA), and triethylamine (TEA), diethanolamine (DEOA), and ethanolamine (EOA) for various irradiation times.

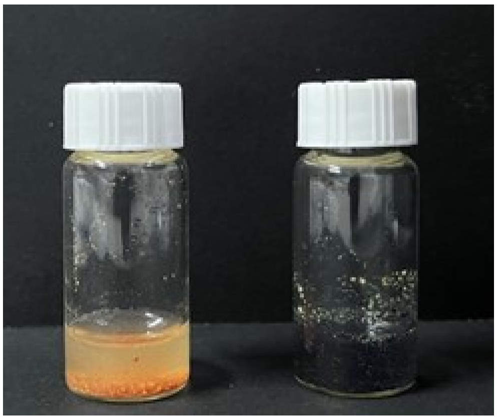

Figure S11. Photography images of the reaction mixture for NADH generation in the presence of triethanolamine (TEOA) and L-ascorbic acid (AA).

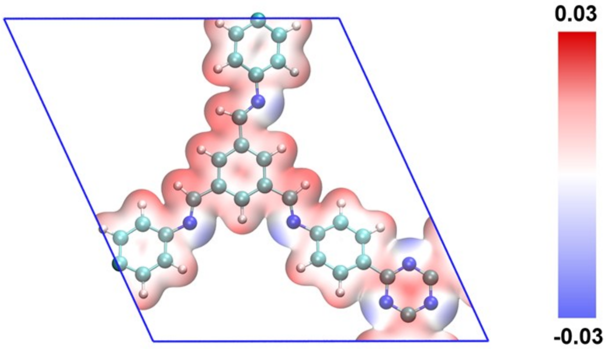

Figure S12. The surface electrostatic potential of TTA-TFB-COF is based on periodic calculation.

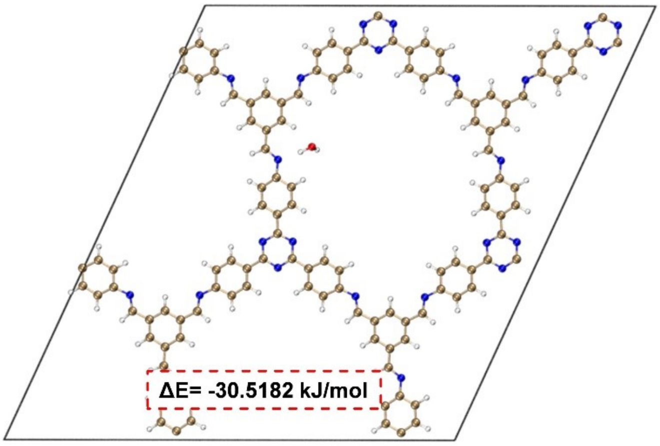

Figure S13. The adsorption energy of TTA-TFB-COF and water.

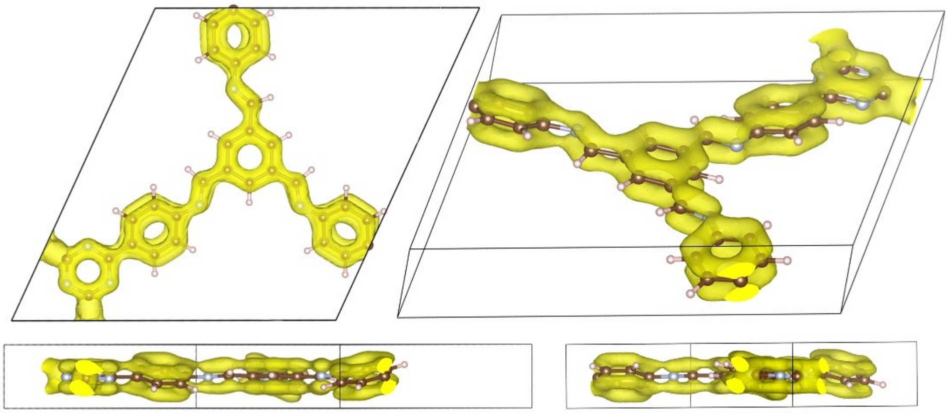

Figure S14. π electronic structure of a fragment of TTA-TFB-COF.
